# Design and Construction of a Novel Humanized Single-Chain Variable-Fragment Antibody Against the Tumor Necrosis Factor Alpha

**Published:** 2019

**Authors:** Davoud Farajzadeh, Sadigheh Karimi-Gharigh, Parisa Jalali-Kondori, Siavoush Dastmalchi

**Affiliations:** a *Department of Cellular and Molecular Biology, Faculty of Basic Sciences, Azarbaijan Shahid Madani University, Tabriz, Iran. *; b *Biotechnology Research Center and Department of Medicinal Chemistry, School of Pharmacy, Tabriz University of Medical Sciences, Tabriz, Iran.*; c *Faculty of Pharmacy, Near East University, Nicosia, North Cyprus, Mersin 10, Turkey*

**Keywords:** TNF-α, Single chain antibody, Affinity chromatography, Pull down, MTT assay

## Abstract

The pro-inflammatory cytokine, TNF-α, which plays a major role in the development and persistence of diseases such as Crohn’s disease, psoriasis, psoriatic arthritis, and rheumatoid arthritis, is the basis for the use of anti-TNF-α therapies. The neutralization of TNF-α or blockage of its binding to the corresponding receptor has mainly served as a therapeutic strategy against some inflammatory diseases. This study aimed to investigate the production of a humanized single chain antibody (scFv) against TNF-α. Therefore, a murine monoclonal antibody, D2 mAb, was selected for humanizing by the complementarity determining region (CDR)-grafting method. Briefly, the replacement of the CDRs from D2 mAb with the specific human single chain scaffold led to the production of a novel humanized single chain fragment variable mAb against human TNF-α (hD2). The subsequent cloning of hD2 into a suitable expression vector, pGEX-6P-1, resulted in the expression of a 52-kDa GST-fusion protein in *E. coli*, mostly in the form of inclusion bodies. The solubilization and refolding of GST-hD2 inclusion bodies was achieved with the addition of 4 M urea and subsequent dialysis to recover the fusion protein in soluble form. Then the soluble GST-hD2 was purified by affinity chromatography through immobilized glutathione. The GST pull-down experiment showed a positive interaction between GST-hD2 and TNF-α protein. Moreover, the results of an MTT assay showed that the purified GST-hD2 has TNF-α neutralizing activity (Kd of 1.03 nM) and hence hD2 has the potential to be developed into a therapeutic agent. However, more investigation is needed to elucidate the potential of *in-vivo* TNF-α neutralizing activity of hD2 in comparison to other anti-TNF-α antibodies.

## Introduction

Tumor necrosis factor alpha (TNF-α) is a pro-inflammatory cytokine, and its abnormal over-expression causes many inflammatory or autoimmune diseases (1). TNF-α neutralizing agents and inhibitors have being used extensively to treat inflammatory diseases such as Crohn’s disease (CD), psoriasis, psoriatic arthritis (PA), and rheumatoid arthritis (RA), which result from the excessive production of TNF-α in the body (2).

Various strategies including blocking of TNF-α mRNA synthesis, inhibition of its post-translational processing, and blocking of the activation of TNF-α receptors have been used to inhibit TNF-α biosynthesis (3). The utilization of monoclonal antibodies (mAbs) or soluble receptors has been reported to be commonly effective in the treatment of several TNF-α-related diseases. Infliximab (Remicade), adalimumab (Humira), golimumab (Simponi), certolizumabpegol (Cimzia), and etanercept (Enbrel), which are all currently approved by the Food and Drug Administration (FDA), are being used in humans to cure TNF-α-mediated diseases (4). 

However, a number of studies have reported some restrictions in the therapeutic uses of TNF-α inhibitors, including low stability, high production expenses, considerable treatment costs, and undesired side effects (5-7), as well as the broadly development of neutralizing antibodies against TNF-α inhibitors in a subset of treated patients. All of these may result in reduced or loss of therapeutic efficacy of these anti-TNF-α agents (8). 

Recently, antibody engineering technology has been used to produce single-chain fragment variable (scFv) antibodies in which the genes encoding for V_H_ and V_L_ are joined together with a short flexible peptide linker (9). Occasionally, scFv antibodies are also manipulated by using the complementarity determining region (CDR) grafting method, replacing the murine content with the amino acid residues of human counterparts to generate a humanized version (10). In comparison with the parental antibody, the humanized scFv antibodies have several advantages in clinical uses, including better tissue penetration, more quick blood clearance, and lower retention times in non-target tissue. They also reduced immunogenicity as much as possible while they could retain the intact antigen binding site and then maintain the specificity and affinity toward the antigen (11).

The need for new TNF-α-blocking agent(s) with higher binding affinity and better neutralizing activity is always sought, because it provides the possibility of using antibodies at lower doses, with less immunogenicity, and for a wider range of patients afflicted with TNF-α-related diseases.

The aim of the present study is to develop a humanized single chain antibody with anti-TNF-α activity suitable for the development of new therapeutic agents useful in inflammatory diseases. 

## Experimental

*Reagents*

PCR master mix, *Bam*HI, and *Eco*RI were provided from Fermentas (Lituania).Acrylamide, N,N′-methylene-bis-acrylamide, 2-mercaptoethanol, and Dimethyl sulfoxide (DMSO) were prepared from Merck (Germany). Tris-HCL, NaCl, yeast extract, Peptone, Isopropyl β-D-Thiogalactopyranoside (IPTG), Agar, Triton X-10, Triton X-100, urea, N,N, N′,N′-tetramethylethylenediamine (TEMED), Dithiothreitol (DTT), phenylmethylsulfonyl fluoride (PMSF), and trypsin were purchased from AppliChem (Germany). Ni-Sepharose 4B was obtained from GE Healthcare Life Sciences (Sweden). Agarose was from Invitrogen (UK). Plasmid mini extraction kit was purchased from Roche (Germany). (3-(4,5-dimethylthiazol-2-yl)-2,5-diphenyltetrazolium bromide (MTT), fetal bovine serum (FBS), and RPMI medium was purchased from Sigma (USA). All chemicals and reagents were of molecular biology grade.

*Selection of anti-TNF alpha monoclonal antibody*

To humanize an efficient murine anti-TNF-α antibody, the selection was carried out based on searching through the known antibodies (Table 1) with high TNF-α neutralizing activity at nM range. 

*Predicting antibody complementarity determining regions*

The IMGT/V-QUEST program (version 3.3.1) was used to predict the complementarity determining regions (CDRs) on both heavy and light chains of the selected antibody (12). In addition, we aligned all published available sequences of human anti-TNF-α scFv antibodies deposited in NCBI databank and then based on the alignment the CDR regions were assigned. 

*Design and Synthesis of human anti-TNF-α scFv encoding gene*

A humanized version of the selected anti-TNF-α scFv (13) was designed based on CDR grafting method. Indeed, to further reduce the immunogenicity of the mouse variable regions, humanized antibodies have been constructed by grafting the complementarity determining regions (CDRs) of a murine mAb onto homologous human antibody variable region. Comparing a large number of human antibody sequences have resulted in a common scaffold for all human antibodies, named Tomlinson scaffold, which have 18 different amino acid positions randomly changed at antigen binding regions, i.e. CDRs. Most of scFv antibody libraries, such as Tomlinson I+J, have established based on the mutations in the positions mentioned above (14). In the present study, we used Tomlinson sequence as a human framework donor and replaced the appropriate CDR coding sequences (responsible for the desired binding properties) of the murine D2 antibody with the corresponding regions of Tomlinson to generate a humanized version of D2, i.e. hD2. The DNA sequence of the gene had been determined by reverse translation of the amino acid sequence of the designed scFv using the codons found in highly expressed *E. coli* genes (15) and taking into account the codon redundancy where appropriate. The sequence was then searched for potential restriction endonuclease sites. Based on the results, two restriction sites (*Bam*HI and *Eco*RI) were introduced at 5′- and 3′-UTR of the fragment, respectively. Sequences were checked to ensure the lack of formation of stem–loop structures and internal sequence similarities. The DNA sequence design was performed using BioEdit software (version 7.0, BioEdit Sequence Alignment Editor Software, Department of Microbiology, North California State University). 

The DNA sequence was synthesized and cloned into pGEX-6P-1 vector by Eurofins Genomics, Germany (http://www.eurofinsgenomics.eu/en/). The received construct was transformed into *E. coli *DH5α for amplification. The amplified DNA was then used to verify the accuracy of the designed scFv encoding gene by PCR, restriction enzyme digestion pattern, and sequencing by Bioneer, South Korea.

*Expression of the synthesized human anti-TNF-α scFv in E. coli*

The recombinant plasmid carrying anti-TNF-α scFv was transformed into *E. coli* BL21 (DE3) cells for the expression of scFv as a GST-fusion protein. The transformants were grown on LB agar supplemented with ampicillin (100 µg mL^-1^ final concentration) at 37 °C overnight and agitated at 180 rpm. The cultures were then diluted 1:100 with fresh LB medium plus antibiotic, and grown to OD_600_ value of 0.6 at 37 °C. The expression of the fusion protein was induced by the addition of IPTG at a concentration of 0.5 mM at 20 °C. The cells were harvested at intervals of 1, 3, 6, and 24 h after induction. The harvested cells were re-suspended in 10 mM Tris-HCl (pH 8.0) and lysed by sonication. The cellular debris was pelleted by centrifugation at 12000 *g* for 15 min. Samples from both supernatant and pellet were analyzed by electrophoresis on a 12% SDS-PAGE under reducing conditions after staining with coomassie brilliant blue (16). 

*In-vitro denaturation and refolding of the inclusion bodies*

The cell lysate was centrifuged at 4 °C for 20 min at 30,000 g. The pellet was then re-suspended in a 10 mL wash buffer (50 mM Tris-HCl pH 7.5, 50-200 mM NaCl) containing 1% Triton X-100 and 1 M urea per gram cell wet weight and incubated at room temperature for 5 min. The cell lysate was centrifuged again at the above-mentioned condition, and the pellet was re-suspended in 10 mL wash buffer. Subsequently, it was centrifuged at 4 °C for 30 minat 15,000 *g*. In the next step, the inclusion bodies (IBs) were re-suspended in the extraction buffer (50 mM Tris-HCl pH 7.5, 4 M urea, 1mM PMSF, and 1mM DTT( at the final protein concentration of 1 mg mL^-1^ and incubated at room temperature for 60 min. Finally, the solution was dialyzed overnight against a 100-fold volume of wash buffer. This contained a gradient of urea concentration and the dialysate was centrifuged at 4 °C for 30 min at 15,000 *g *(17, 18). 

*Affinity purification of GST-hD2*

Purification of the refolded fusion protein was achieved using Glutathione Sepharose 4B bulk matrix (GE Healthcare) according to the manufacturer′s instructions (19).

*Pull down assay*

The TNF-α (the probe) and GST-hD2 fusion proteins (the target) are incubated together with glutathione-agarose beads and then the complex (TNF-α-GST-hD2) was recovered from the beads and analyzed using SDS-PAGE experiment. Briefly, 25 µg of the fusion protein was incubated with 25 µg of TNF-α and 50 µL of a 50% slurry of glutathione-agarose beads previously equilibrated with equilibration buffer (50 mM Tris (pH 8.0), 100 mM NaCl, 1.4 mM PMSF, 0.1% 2-mercaptoethanol, and 1% Triton X100) for 2 h at 4 °C, while mixing by inverting at cold room. The mixture was centrifuged at 13,000 *g* for 2 min at 4 °C, and the supernatant was discarded. Then, beads were washed four times with 1 mL of ice-cold GST wash buffer consist of 50 mM Tris (pH 8.0), 100 mM NaCl and 0.1% 2-mercaptoethanol and then were centrifuged as before for 1 min at 4 °C and the supernatant was discarded. Samples of beads from each step were collected and analyzed by SDS-PAGE to determine the association between the fusion protein and TNF-α (20). 

*MTT assay*

100 µL per well of murine fibroblast L929 cells in RPMI medium supplemented with 10% fetal bovine serum (FBS) were seeded in 96-well plates at 1×10^5^ cells mL^-1^ and incubated for 20 h. Also several dilutions of GST- hD2 were prepared in medium containing actinomycin D (10 µg mL^-1^) and TNF-α (2 ng mL^-1^) and incubated at 37 °C for 2 h. After removing of the supernatants of the cultured L929 cells, different concentrations of GST- hD2 were added to the wells. Then the cells were incubated at 37 °C for 24 h, and the supernatants were removed again. To each well, MTT at 5 mg mL^-1^ concentration was added and incubation was continued at room temperature for 4 h. After removing supernatant, the solubilization buffer (Sorensen buffer 12.5% and DMSO 87.5%) was added to each well with shaking for 40 min at 25 °C. The plate was read in ELISA Reader for measuring OD in 570 nm (background was read at 630 nm wavelength) (21). Blank control (culture alone), TNF-α control (TNF-α alone), and antibody control (hD2 alone) were also included in the experiment. 

## Results

*Selection of anti-TNF-α mAb and determining its CDR regions*

**Figure 1:**
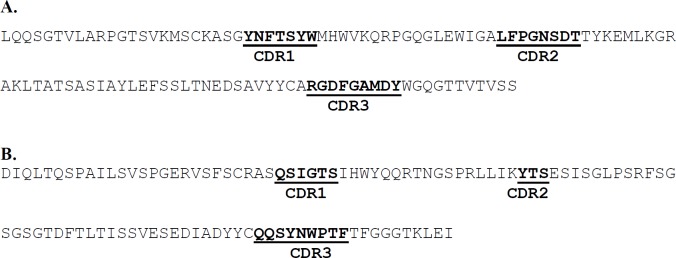
Prediction of complementarity determining regions (CDRs) of anti-human TNF-α D2 immunoglobulin heavy and light chain variable regions using the IMGT/V-QUEST programme. A and B represent the heavy and light chain variable regions, respectively

**Figure 2:**
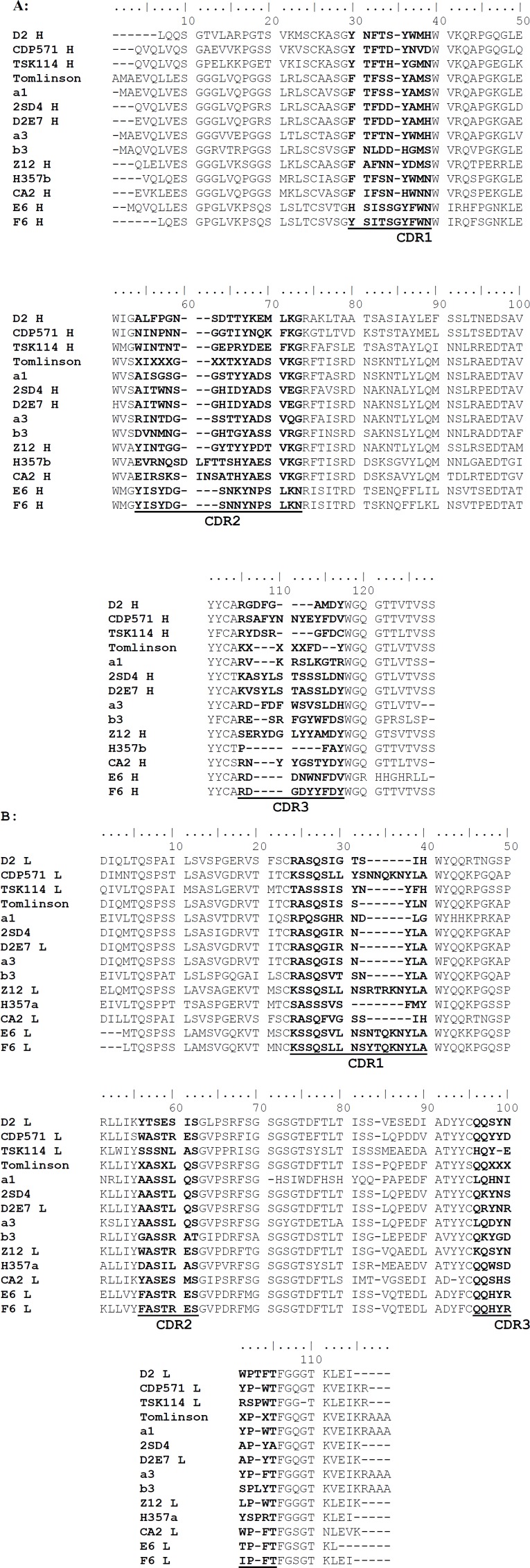
Determination of CDRs of anti-human TNF-α D2 immunoglobulin heavy and light chain variable regions based on aligning the published available sequences of human anti-TNF-α scFv antibodies. A and B represent the heavy and light chain variable regions, respectively

As the aim of the current study was to prepare a humanized scFv antibody against TNF-α, the candidate antibodies were limited to those which were not originated from human. On the other hand, the strategy adopted in this work was to make a decision based on TNF-α neutralizing activity for the antibodies observed in biological assay. Table 1 shows a list of different types of antibodies developed against TNF-α from different murine and human sources. According to the table, murine D2 (mD2) single chain antibody was selected for humanization based on its high TNF-α neutralizing activity (with Kd at nM range). The human antibodies, and those previously humanized and/or commercialized were excluded from investigation (i.e., 2SD4 (22), D2E7 (22), CDP571 (Humicade), Z12 (23), m357 (24), and CA2 (Inflximab) (25)). TSK114 (26) was also excluded as its binding affinity to TNF-α was measured by surface plasmon resonance technique (SPR) which is not a method based on biological end point. We predicted the CDR regions on both heavy and light chains of the mD2 antibody using the IMGT/V-QUEST tool. Three CDRs for each of the heavy and light chains were determined as shown in Figure 1. Similarly, the alignment of the sequences of the known anti-TNF-α scFv mAbs (Figure 2) revealed almost the same regions as the CDRs. As shown in figure 2, a big portion of the aligned sequences is related to the conserved framework regions of the antibodies and the remaining segments are CDR1, CDR2 and CDR3 regions in both light and heavy chains, respectively.

**Table 1:** Characteristics of the published available anti-TNF-α antibodies

**Anti-TNF-α antibody**	**Type**	***Assay***	***K*** **d (M)**	**Reference**
D2	Murine monoclonal	MTT	0.86 × 10-9	Zhu *et al*. 2006 (13)
CDP571(Humicade)	Humanized scFv	nd	8.7 × 10-11	Absolute Antibody
TSK114	Murine monoclonal	SPR	~5.3 × 10-12	Song *et al. *2008 (26)
2SD4	Fully human monoclonal	SPR	0.20 × 10-10	Santora *et al*. 2001 (22)
D2E7	Fully human monoclonal	SPR	0.1 × 10-11	Santora *et al*. 2001 (22)
Z12	Murine monoclonal	ELISA	0.1×10-9	Qin *et al. *2006 (38)
m357	Murine monoclonal	MTT	5.5 × 10-9	Chiu *et al*. 2011 (24)
CA2 (Infliximab)	mouse-human chimeric antibody	Cell binding	0.46 × 10-10	Scallon *et al*. 1995 (39)
E6	Murine monoclonal	MTT	1.7× 10-9	Zhu *et al*. 2006 (13)
F6	Murine monoclonal	MTT	1.7× 10-9	Zhu *et al*. 2006 (13)

nd, not determined; SPR, Surface plasmon resonance technique; Cell binding, Membrane TNF-α binding affinity assay.

The Kd value has been reported by the provider (Absolute Antibody) viewed at http://absoluteantibody.com/ab/Anti-TNF-alpha-CDP-

571-Humicade/

**Figure 3:**
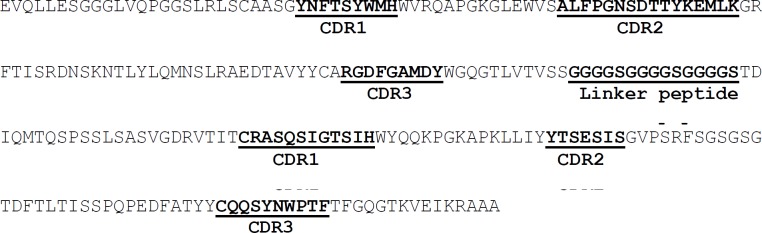
Amino acid sequence of humanized version of anti-TNF-α scFv hD2. VH and VL regions were linked into a single molecule via a short ﬂexible linker peptide (Gly_4_Ser)_3_

**Figure 4:**
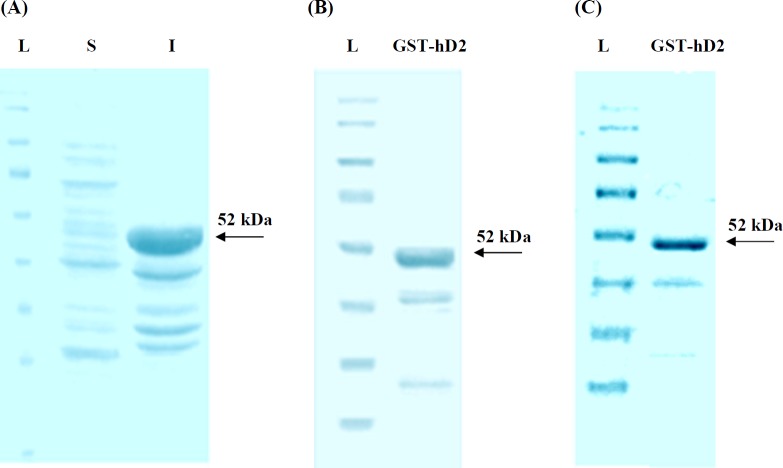
SDS-PAGE analysis of the synthesized human anti-TNF-α scFv expression in *E. coli*. (**A**) L, S and I represent protein molecular size marker (SM0671), supernatant and cellular debris of bacteria, respectively; 3h after induction by IPTG; (B) SDS-PAGE analysis of GST-hD2 fusion protein. The purified soluble GST-hD2 after solubilization of inclusion bodies; (C) Purified soluble GST-hD2 protein resulted from glutathione affinity purification

**Figure 5:**
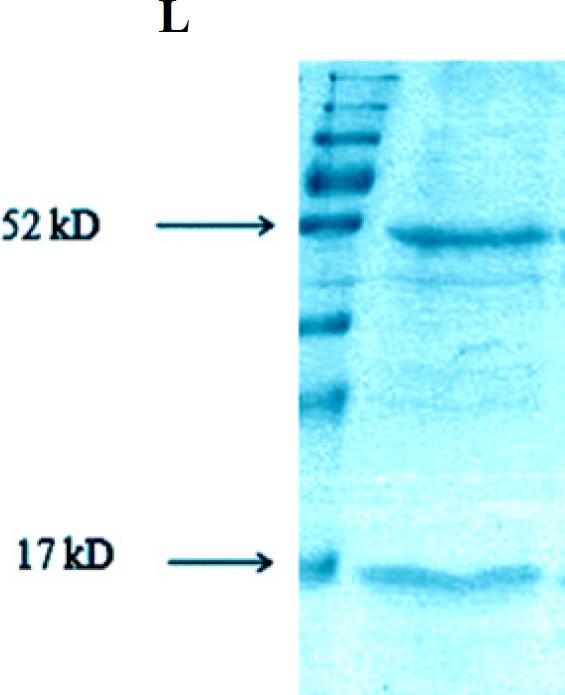
Analysis of interaction between TNF-α L and GST-hD2 fusion protein

L represents protein molecular size marker (SM0671). Both TNF-α and GST-hD2 proteins are detectable with the expected molecular weights of 17 and 52-kDa, respectively, indicating an interaction between TNF-α and fusion protein GST-hD2 protein.

**Figure 6:**
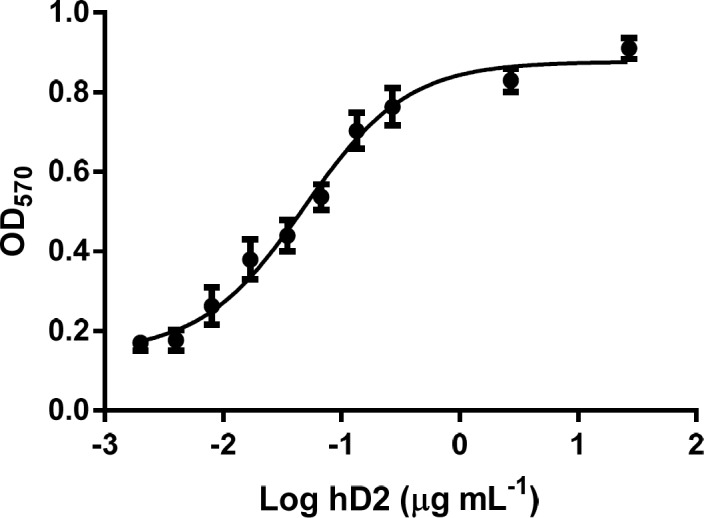
Neutralization of TNF-α-mediated cytotoxicity in L929 cells by the GST-hD2. The survival of L929 cells treated with 0 to 27 µg mL^-1^ of hD2 scFv antibody, in the presence of TNF-α (2 ng mL^-1^) was determined by MTT assay. Twenty seven micrograms per milliliter of hD2 could completely neutralize TNF-α-mediated cytotoxicity in L929 cells. Error bares represent standard deviations calculated from data of three experimental replicates

*Design, Synthesis and Expression of hD2 encoding gene*

In order to construct the humanized D2 scFv, CDR grafting technique was used. To this end the sequences of CDRs from the mD2 mAb were used to replace the CDRs of the specific human single chain framework represented by Tomlinson I+J sequence (Figure 3). The designed DNA sequence (intended to encode human D2 scFv antibody) was synthesized and cloned into pGEX-6P-1 vector by Eurofins Genomics and called pGEXhD2. The results of PCR, digestion pattern and sequencing confirmed the accuracy of the hD2 coding gene inserted into pGEXhD2 construct. The sequencing showed that the size of the encoding region was 726 bp, composed of 366, 45, and 378 bp segments encoding the variable heavy chain, a (Gly_4_Ser)_3_ linker, and variable light chain, respectively as expected.

The transformation of pGEXhD2 into *E. coli* BL21 (DE3) cells was produced recombinant bacterial colonies which were resistant to ampicillin and capable to express GST-hD2 fusion protein. The SDS-

*Pull down assay*

The result of pull down experiment was shown in Figure 5. Both TNF-α and GST-hD2 proteins are detectable with the expected molecular weights of 17 and 52-kDa, respectively, indicating an interaction between TNF-α and fusion protein GST-hD2 protein.

*TNF-α cytotoxicity neutralizing activity of purified hD2 scFv*

MTT assay was applied to determine the cell survival of L929 cells which were treated with 0 to 27 µg mL^-1^ of hD2 scFv antibody, in the presence of TNF-α (2 ng mL^-1^) for 24 h. The results showed that the L929 cells death was completely occurred at 2 ng mL^-1 ^of TNF-α. Twenty seven micrograms per milliliter of hD2 could completely neutralize TNF-α (2 ng mL^-1^) mediated cytotoxicity in L929 cells. The results showed that anti-TNF-α hD2 scFv protects cells form TNF-α cytotoxicity in a dose-dependent manner (Figure 6) with Kd value of 1.03 nM. Antibody alone (anti-TNF-α hD2 scFv) did not show any significant effect on the survival of L929 cells. Statistical parameters such as mean and standard deviation were calculated from triplicate data.

## Discussion

A number of studies have reported some restrictions in the therapeutic uses of anti-TNF-α antibodies, including low stability, high production expenses, considerable treatment costs, and undesired side effects (5-7), as well as the broadly development of neutralizing antibodies in a subset of patients treated with anti-TNF-α antibodies. All of these may result in reduced or loss of therapeutic efficacy of these anti-TNF-α agents (8). Therefore, the use of considerably smaller genetically engineered antibody fragments has been shown to be a useful approach to resolve such problems (27, 28). In this regard, even though humanized scFv antibody molecules have only one-sixth the molecular mass of the intact IgG antibodies, in comparison with the parental antibody, they have several advantages in clinical uses, including the possibility of using at lower doses, better tissue penetration, more quick blood clearance, and lower retention times in non-target tissue. Also, previous studies have demonstrated that scFv antibodies have lower immunogenicity than intact antibodies while retaining the intact antigen binding site, the specificity, and affinity toward the antigen for a wider range of patients afflicted with TNF-α-related diseases (11, 29-31). By contrast to the full length antibodies that are produced mostly in mammalian cells, the scFvs can be produced easily on large scales with much less expenses in microbial hosts such as *E. coli *(32).

The aim of this study was to design a humanized scFv antibody against TNF-α with high affinity and low immunogenicity applicable in the development of therapeutic and diagnostic agents. Therefore, among the available ten scFv murine antibodies listed in Table 1, the murine D2 (mD2) scFv antibody was selected to be humanized based on its high neutralizing activity against TNF-α. The CDR regions were predicted on both heavy and light chains of the mD2 antibody based on aligning its sequence with other anti-TNF-α scFv antibodies, as well as analyzing the mD2 sequence based on the knowledge-based algorithms implemented in the IMGT/V-QUEST program (12). The results of both multiple sequence alignment and sequence analysis by software were in close agreement and we have designed our humanized D2 using the identified CDRs of mD2 using the former approach. Although the software algorithm is based on the sequence alignment of the query with the reference sequences in the database, our multiple alignment was generated among scFv antibodies known to recognize a single target-that is, TNF-α. Thus, we concluded that determining CDRs, based on aligning their sequences, was an efficient method that could be used in our study. To conclude, one may claim that the sequences of antibodies are so similar in their framework that the sequence variations are found only in the CDR regions; therefore, the regions with low homology can be easily determined by aligning the antibody sequences (33, 34).

CDR-grafting technique is a well-known method for constructing novel antibodies that have improved characteristics (35). The most important application of this method is to humanize high-affinity antibodies developed in other species by inserting the appropriate CDR-coding segments into a human antibody scaffold (34, 36). In one study, the variable domain-resurfacing approach was employed to humanize the murine monoclonal antibody m357. They showed that the purified h357 antibody was capable of maintaining the high antigen-binding affinity and inhibiting disease progression significantly in a mouse model of antibody-induced arthritis in collagen (24). 

In the present study, the CDRs from the mD2 anti-TNF-α scFv antibody were used to replace the CDRs of the specific human’s single-chain framework (Tomlinson I+J library scaffold) to develop a humanized version of mD2 scFv (named hD2) with the sequence shown in Figure 3. Synthesis, cloning, and expression of hD2 scFv in reducing bacterial cytoplasm led to the forming of insoluble inclusion bodies, which were solubilized and refolded—by the adding and gradual removal of a denaturing agent (i.e., 4 M urea) —to ultimately obtain the properly folded and functional hD2. Many proteins fail to fold properly during the recombinant protein expression in *E. coli. *Therefore, the protein ends up as aggregates found in the inclusion bodies. This may be due to fast overexpression, absence of chaperones, or unfavorable oxidizing of the intracellular environment of the prokaryotic cell. Denaturing with chaotropic agents, such as 8 M urea or 6 M guanidine HCl, and then refolding in favorable conditions is a common technique used for isolating soluble-folded proteins from inclusion bodies (37).

In this work, salts and unwanted proteins of the host cell were eliminated by a two-step wash procedure. Removing these impurities and obtaining the IBs with high recovery and purity is required because impurities interfere with the refolding and significantly affect the yield and purity of the process. We used 1% Triton X-100 to solubilize the components of the bacterial cell wall that contaminate the inclusion-body preparation, and we used 1 M urea to remove any residual cell debris. In addition, we found that this method provided the most effective means for the refolding of aggregated proteins when the refolding procedure was carried out with a gradually decreasing concentration of urea in the extraction buffer. Thus, optimization of the early stages of the downstream process will impact the yield of the overall process and the purity of the final product. 

Glutathione-S-transferase (GST) fusion proteins have a range of applications in the detection, isolation and purification of recombinant proteins. A GST pull-down experiment also is used to confirm suspected interactions between a probe protein and a known protein. In the present study, the pull down assay showed the positive interaction between GST-hD2 and TNF-α, suggesting an affinity between the bait and predator proteins. Moreover, the results of an MTT assay showed that the purified GST-hD2 has TNF-α neutralizing activity and hence hD2 may be regarded as a promising antibody to be converted into a therapeutic agent. However, more investigation is needed to elucidate more precisely the potential of *in-vivo *TNF-α neutralizing activity of hD2 compared to other anti-TNF-α antibodies.
